# Decomposition and comparative analysis of the differences in anxiety symptoms in preschool children with only and non-only children: a cross-sectional study

**DOI:** 10.3389/fpsyt.2026.1877032

**Published:** 2026-10-14

**Authors:** Yuying Xie, Wenyu Wang, Yusupujiang Tuersun, Xiaona Yin, Qiang Zhou, Zhenning Liang, Shasha Peng, Xun Li, Junfang Huang

**Affiliations:** 1 Department of Pediatrics, Shenzhen Longhua District Maternity and Child Health Hospital, Shenzhen, Guangdong, China; 2 School of Public Health, Southern Medical University, Guangzhou, Guangdong, China; 3 School of Health Management, Southern Medical University, Guangzhou, Guangdong, China; 4 Drug and Cosmetics Supervision and Administration Section, Lianyungang Market Supervision Administration, Lianyungang, Jiangsu, China; 5 Shenzhen Longhua Maternity and Child Healthcare Hospital, Shenzhen, Guangdong, China; 6 The Seventh Affiliated Hospital, Sun Yat-sen University, Shenzhen, Guangdong, China; 7 School of Public Health, Sun Yat-sen University, Guangzhou, Guangdong, China

**Keywords:** anxiety symptoms, cross-sectional study, Fairlie decomposition model, only child, preschool children

## Abstract

**Background:**

Against the backdrop of family structure transformation and increasing attention to child mental health, anxiety symptoms in preschool children have become an important public health issue. Anxiety symptoms not only affect early emotional regulation and social adaptation but may also have sustained impacts on subsequent psychological development. In the context of China’s unique population policy, significant differences exist between only children and non-only children in terms of family resource allocation, parent-child interactions, and developmental environment, which may further influence the manifestation of childhood anxiety symptoms.

**Objective:**

This study aims to compare the differences in the detection rates of anxiety symptoms between only-child and non-only-child preschool children, identify the differential effects of individual, family, and neurobehavioral factors on the two groups, and quantify the contributions of various factors to between-group differences using the Fairlie decomposition model, thereby providing empirical evidence for targeted child mental health intervention strategies.

**Methods:**

This cross-sectional study was based on data from the 2022 Longhua Child Cohort Study in Shenzhen, China. A total of 67,779 preschool children aged 3–6 years (46,565 only children; 21,214 non-only children) were included and divided into two groups according to only-child status. Anxiety symptoms were assessed using the Chinese version of the Spence Preschool Anxiety Scale (SPAS, cutoff ≥48). Sociodemographic characteristics, ADHD symptoms, and emotional and behavioral problems were also collected. Chi-square tests and multivariate logistic regression were used to analyze associated factors, and the Fairlie decomposition model was applied to decompose between-group differences and quantify the contribution of each factor to the disparity in anxiety symptoms.

**Results:**

The overall detection rate of anxiety symptoms in preschool children was 3.48%, with only-children showing a slightly higher rate (3.6%) than non-only children (3.3%, P = 0.027). Although the difference reached statistical significance, the absolute magnitude of the difference was small. Multivariate logistic regression revealed that higher maternal education level (OR = 0.710–0.751) and higher household income (OR = 0.426–0.623) were both associated with a decreased risk of anxiety symptoms. In contrast, female sex (OR = 1.224–1.276), positive ADHD symptoms (OR≈2.53), and abnormal SDQ total difficulties scores (OR≈18.28) were associated with an increased risk of anxiety, serving as risk factors for anxiety symptoms. Fairlie decomposition results showed that 39.8% of the total difference in anxiety symptoms between the two groups could be explained by observed variables, with emotional and behavioral problems contributing the most (41.43%), followed by ADHD symptoms (17.93%) and gender (3.42%). Meanwhile, maternal education level and household income exhibited significant negative contributions (-19.67%and-5.91%, respectively), suggesting that higher socioeconomic status served as a “protective buffer” against anxiety risk for only children. The remaining 60.2% of the difference may be attributable to unobserved factors.

**Conclusion:**

There was a statistically significant difference in the detection rate of anxiety symptoms between only-child and non-only-child preschool children. This disparity was influenced by multiple factors, including children’s emotional and behavioral problems, ADHD symptoms, and family socioeconomic factors. However, the practical significance of this difference still requires further interpretation in conjunction with additional family environmental variables. In light of the differing mental health needs of children from diverse family structures, this study recommends establishing a mental health monitoring system centered on SDQ screening, implementing differentiated family education guidance tailored to different family structures, and promoting a collaborative mental health support system involving kindergartens, families, and communities.

## 1 Introduction

Anxiety symptoms in preschool children have become an increasingly serious public health issue worldwide (1, 2). Anxiety symptoms are not only associated with children’s emotional status and social competence, but may also affect their subsequent psychological development and functional performance. Studies indicate that the prevalence of anxiety symptoms among preschool children is relatively high, particularly in regions experiencing rapid urbanization. Recent research shows that the incidence of anxiety symptoms in preschool children ranges between 10% and 20% (3), and can even be as high as 34.2% in some areas (4). Differences in findings across studies may be attributed to variations in study populations, regional development levels, assessment instruments, and diagnostic criteria, and also suggest that children’s mental health status may be jointly influenced by multiple factors such as social environment and family background. The persistence of anxiety symptoms during early childhood may be associated with subsequent mental health problems. Previous studies have indicated that anxiety symptoms emerging in the preschool period may persist into school age and are associated with other mental health manifestations such as depressive symptoms and behavioral problems (5). Therefore, identifying the factors associated with anxiety symptoms in preschool children and exploring the differences in children’s mental health outcomes across different individual and family backgrounds are of great significance for improving early mental health screening and support strategies.

Among the many social and family factors influencing anxiety symptoms in preschool children, family structure is a significant factor that cannot be ignored. Family structure refers to the number and composition of family members, as well as the relationships and interaction patterns among them. It involves not only the number and composition of family members but also relates to parenting styles, parent-child interactions, and the support system within the family. Existing research indicates that differences in family structure may significantly impact children’s emotional health, particularly in the manifestation of anxiety symptoms (6). Studies have found that children from single-parent families, reconstituted families, and high-conflict families are more likely to experience emotional problems, especially anxiety symptoms (7). In contrast, children from two-parent families, who receive more emotional support and a stable family environment, generally exhibit more ideal mental health conditions (8). In China, the implementation of the one-child policy has made only children a unique social group. Compared with non-only children, only children lack the companionship and interaction of siblings at home, and their upbringing is primarily parent-led, which may expose them to distinct challenges regarding emotional attention, educational expectations, and psychological pressure (9) Therefore, gaining a deeper understanding of the differences in anxiety symptoms across different family structures, particularly between only children and non-only children, not only helps reveal the impact of the family environment on children’s mental health but also provides a valuable theoretical basis for future intervention strategies and policy formulation.

Numerous studies have been reported on anxiety symptoms in children, which typically manifest in various forms such as excessive worry, social fear, and separation anxiety. Previous studies have indicated that family environments differ across various family structures, and these differences may be associated with children’s emotional health status (10). Children from single-parent families, reconstituted families, and high-conflict families are more likely to exhibit emotional problems, whereas a stable family environment and positive family support are generally associated with better child mental health outcomes (3). In China, only-children differ from non-only-children in terms of family membership composition, sibling interaction, and patterns of parental investment, and these differences may be related to children’s emotional and behavioral outcomes. However, the influence of family structure on child mental health is not unidimensional; only-children may receive more family resources and parental attention, while also facing different educational expectations and family interaction patterns. Therefore, comparing the differences in anxiety symptoms between only-children and non-only-children may help to further understand the characteristics of child mental health across different family structures and provide references for early mental health support (11).

Although extensive research exists on anxiety symptoms in preschool children, studies on the differences in anxiety symptoms between only children and non-only children remain relatively scarce. Particularly in the context of ongoing changes in family structure in China, the mental health characteristics of children from different family structures and their associated factors still warrant further exploration. The present study aims to compare the differences in anxiety symptoms between only-child and non-only-child preschool children, and to further analyze the associations between family socioeconomic factors, other related factors, and anxiety symptoms. It is important to emphasize that the relationship between socioeconomic status and child mental health may be complex. On one hand, families with higher socioeconomic status can provide more abundant educational resources, healthcare services, and family support, which may contribute to better child mental health outcomes. On the other hand, higher socioeconomic status may also be accompanied by heightened educational expectations and greater parental investment, which may increase psychological pressure on children. Therefore, the association between socioeconomic status and child anxiety symptoms may be jointly influenced by factors such as parenting styles and the quality of parent–child interactions.

## 2 Methods

### 2.1 Data source

The participants were recruited from the 2022 LCCS survey. The LCCS is an ongoing prospective cohort study of preschoolers in the Longhua District, Shenzhen, China. In 2022, a questionnaire survey was distributed among 250 kindergarten parents in Longhua District, Shenzhen, China. Exclusion criteria included children with severe physical illnesses or mental disorders. A total of 75,557 questionnaires were distributed, and 71,069 questionnaires were returned, with a recovery rate of 94.06%. After excluding participants with missing or invalid data on study variables, the final analysis included 67,779 children (**Supplementary Figure 1**). This study was approved by the Ethics Committee of Shenzhen Longhua Maternal and Child Health Hospital (ethics license number: 2016102501) and the School of Public Health, Sun Yat-sen University (ethics license number: 2015–016). Informed consent was obtained from all the study participants.

Kindergarten principals and doctors were trained in the use of questionnaires. The invitation object was extended to the parents of the young children. After parental informed consent was obtained, a questionnaire was administered. This survey used an electronic questionnaire. The questionnaire was used to collect information on anxiety status, ADHD status, emotional and behavior problem status, general information of children and parental socio-demographic characteristics (**Supplementary Figure 1**).

### 2.2 Anxiety symptoms

Anxiety symptoms were measured using the Chinese version of the Spence Preschool Anxiety Scale (SPAS) Chinese version was specifically designed by Chinese researchers to measure anxiety symptoms in preschoolers aged 3–6 years. The scale has good reliability and validity, with a Cronbach’s α coefficient of 0.950 (11). The scale has a clear factor structure, good internal consistency and reasonable criterion validity. There is a strong correlation between the scale and other anxiety symptom scales. The 5-point Likert scoring method was used, with “never” = 0, “rarely”= 1, “sometimes” = 2, “often”= 3, and “always”= 4. The total score of each dimension was obtained by adding the scores of each dimension and all dimensions to obtain the total anxiety score ranging from 0 to 112. Higher scores indicate serious anxiety symptoms. A total score of 48 was defined as positive screening for anxiety symptoms overall Cronbach’s alpha coefficient for this study was 0.897, 0.903 for the group of only-child preschoolers and 0.881 for the group of non-only child preschoolers.

### 2.3 Grouping variables

Based on the reported status as an only child during the survey, respondents were divided into two groups: only child or non-only child.

### 2.4 Covariates

To obtain more reliable results, we controlled for a range of potentially confounding variables. These characteristics include the child general information, parental sociodemographic characteristics, ADHD status in preschool children, and the presence of emotional and behavioural problems, variables that have previously been used in other studies on anxiety symptom status.

### 2.5 Demographic characteristics

Participants were classified by two variables: gender (male or female).

### 2.6 Parental sociodemographic characteristics

The parental education level of preschool children was divided into four groups: Junior high school or lower, High school, College, and Master’s degree or above. The monthly per capita household income is divided into five categories: less than 10,000RMB, 10,000-20,000RMB, 20,000-30,000RMB, 30,000-40,000RMB and more than 40,000RMB.

### 2.7 ADHD status

ADHD symptoms were measured using the 26-item Swanson, Nolan, and Pelham (SNAP) Revision 4 (SNAP-IV) scale, which has been validated in the Chinese population and is widely used for assessing ADHD symptoms (12). The SNAP-IV scale includes two subsets of ADHD symptoms (inattention and hyperactivity/impulsivity) based on DSM-IV criteria, as well as a subset for opposition/defiance. Each item is scored from ‘0’ (not at all) to ‘3’ (very often). Subset scores were calculated by summing the item scores, and children were classified as having borderline problems in inattention or hyperactivity/impulsivity if their respective subset score exceeded 13.The SNAP-IV scale demonstrated excellent reliability with a Cronbach’s α coefficient of 0.929 in this study.

### 2.8 Emotional and behavioural problems

Emotional and behavioural problems in preschool children were investigated using the Strengths and Difficulties Questionnaire (SDQ).The SDQ was developed by Robert Goodman in 1997 and is a commonly used tool for assessing the mental health of children and adolescents (13). The SDQ is a short behavioural screening tool with good psycho measurement properties for detecting mental health problems and psychiatric disorders, it is easily completed by different informants (i.e. patients or relatives) and is user friendly due to its positive attributes and it allows for comparisons to be made between different populations (14). The SDQ consists of five scales with five items per scale. These scales assess emotional symptoms, behavioural problems, hyperactivity/inattention, peer relationships and pro-social behavior. Responses are on a three-point Likert scale: ‘Not true’, ‘somewhat true’ or ‘certainly true’. Negative items were scored from 0 to 2, while positive items were reverse scored from 2 to 0, with higher scores indicating more severe problems. Each subscale is scored on a scale of 0 to 10. The pro-social behavior subscale is assessed independently and is not included in the total score for emotional and behavioral problems. Scores from the first four subscales were summed to obtain a total difficulty score ranging from 0 to 40 (15). Higher total difficulty scores reflect more problems and indicate an increased risk of a psychiatric diagnosis (16). scores of 0–13 are considered normal, scores of 14–16 are considered to be at borderline levels of behavioural and emotional problems, and scores of 17–40 are considered to be abnormal (17).The overall Cronbach’s alpha coefficient for the SDQ in this study was 0.720.

### 2.9 Statistical analysis

Basic demographic characteristics and lifestyle of the study population were analyzed in a descriptive fashion. Continuous variables are described by mean ± SD, while categorical variables are described by percentages. The chi-square test was used to analyze the distribution of anxiety symptoms in only children and non-only children. Subsequently, a binary logistic regression model was constructed in this study to identify the main determinants of anxiety symptoms in only and non-only children. The outcome variables were defined as a binary classification (yes/no) of anxiety symptoms, while independent variables were selected from four domains: child general information, parental sociodemographic characteristics, ADHD status in preschool children, and emotional and behavioral problems. Initially, these variables were screened by univariate analysis (retention criteria: P<0.05), and then, they were included in the multivariate model. Through this process, statistically significant predictors (P<0.05) were retained in the final model. The above statistical analyses were performed using the IBM SPSS Statistics 23 software. Ultimately, the Fairley model was used to examine factors influencing differences in anxiety symptoms in only children and non-only children preschool children. The analysis was performed using the Stata MP 18.0 software. The statistical significance level was set at 0.05.

### 2.10 Fairlie decomposition model

In this study, we applied a multivariate Fairlie decomposition analysis (FDA) based on a binary regression model. FDA is one of the decomposition techniques used in multivariate models to quantify the contribution of predicted differences between two groups to outcome variables. The method is an extension of the Blinder-Oaxaca decomposition analysis, which has been widely criticized for its inefficiency in handling the logit and probit model (18). The FDA was developed specifically for nonlinear regression models, including logit and probit models (19). The FDA identifies the contribution of independent variables to explaining differences between groups by calculating the change in the mean predicted probability resulting from substituting one independent variable at a time in one group (e.g., Group A-preschool children with only children) while holding other variables constant in the other group (e.g., Group B-preschool children with non-only children). The Fairlie decomposition technique works by limiting the predicted probability to between 0 and 1.Studies have shown that the FDA can better quantify the contribution and significance levels of different variables for nonlinear regression models (20, 21). Given that the dependent variable was a dichotomous variable, we employed Fairlie’s nonlinear decomposition to decompose the discrepancies in preschool children’ anxiety symptoms into the contributions of various factors. As outlined by Fairlie (22), the decomposition of the nonlinear equation can be expressed as follows:

(1) $${\bar{Y}}_{e}-{\bar{Y}}_{w}=\left[\sum_{i=1}^{N^{e}}\frac{F\left(X_{i}^{e}\beta^{e}\right)}{N^{e}}-\sum_{i=1}^{N^{w}}\frac{F\left(X_{i}^{w}\beta^{e}\right)}{N^{w}}\right]+\left[\sum_{i=1}^{N^{w}}\frac{F\left(X_{i}^{w}\beta^{e}\right)}{N^{w}}-\sum_{i=1}^{N^{w}}\frac{F\left(X_{i}^{w}\beta^{w}\right)}{N^{w}}\right]$$

The symbols ${\bar{Y}}_{e}$ and ${\bar{Y}}_{w}$ represent the mean probabilities of the two binary outcomes. The F is employed to indicate the cumulative distribution function of the logistic distribution. Ye-Yw represents the total difference in the differences between the two groups. The N^e^ and N^w^ represent the sample sizes of the two samples. The first term in parentheses in (Equation 1) represents the portion of the gap attributable to differences in observed characteristics, while the second term denotes the portion of the gap due to differences in estimated coefficients.

## 3 Results

### 3.1 Basic characteristics of the study subjects

This study included a total of 67,779 samples. The mean SPAS score for the preschool children was 23.110 ± 12.318 (**Supplementary Table 1**). A score below 48 indicated the absence of anxiety symptoms, while a score of 48 or above indicated the presence of anxiety symptoms. The results from **Supplementary Table 2** showed that 65,418 preschool children (96.52%) did not have anxiety symptoms, while 2,361 preschool children (3.48%) were identified as having anxiety symptoms.

The results of the chi-square test in **Supplementary Table 2** revealed that there were significant differences in the distribution of the following seven variables between only-child and non-only child preschool children: Gender, Maternal education, Paternal education, Monthly per capita household income, ADHD symptoms, SDQ classification, and SPAS classification (**Supplementary Tables 1, 2**).

### 3.2 Comparison of variables’ distribution

**Supplementary Table 3** shows the relationship between the distribution of covariates and the presence or absence of anxiety symptoms in only-child and non-only child preschool children. The data indicate that the covariates of Maternal education and Paternal education exhibited similar distribution patterns among preschool children regardless of their anxiety symptom status (**Supplementary Table 3**).

### 3.3 Logistic model results

Multivariate logistic regression analysis revealed distinct patterns of factors influencing anxiety symptoms in only children and non-only children.

In the only-child group, higher maternal education level was significantly associated with a lower risk of anxiety symptoms. Compared to children whose mothers had an education level of junior high school or lower, those whose mothers had a high school education (OR = 0.751, P<0.01) or a college education (OR = 0.710, P<0.01) exhibited significantly lower anxiety risk.

In the non-only child group, the association between household income and reduced anxiety risk was more prominent. Compared to the lowest income group (<10,000 RMB), all subgroups with a monthly per capita household income above 10,000 RMB showed significantly lower anxiety risk (P<0.05), with the strongest association observed in the 30,000–40,000 RMB group (OR = 0.426, P<0.001).

In addition, several common factors were identified across both groups. Female sex was a risk factor for anxiety symptoms in both only-children (OR = 1.224, P<0.001) and non-only-children (OR = 1.276, P<0.01). Meanwhile, the absence of ADHD symptoms was associated with a lower risk of anxiety in both only-children (OR = 0.378, P<0.001) and non-only-children (OR = 0.423, P<0.001). Elevated SDQ total difficulties scores were associated with an increased risk of anxiety in a dose–response manner, with the highest risk observed in the abnormal group (17–40 points), showing a consistent trend in both only-children (OR = 19.586, P<0.001) and non-only-children (OR = 15.314, P<0.001).

In summary, the factors associated with anxiety symptoms in preschool children differed by family structure. Maternal education level was associated with reduced anxiety risk in only-children, whereas household income showed a more pronounced association with reduced anxiety risk in non-only-children. These findings suggest an association between family structure and the manifestation of anxiety symptoms in children (**Supplementary Table 4**).

### 3.4 Decomposition analysis results

To enhance the robustness of the results, this study employed the Fairlie decomposition model to analyze the differences in anxiety symptoms between only children and non-only children. As shown in **Supplementary Table 5**, the total difference in anxiety symptoms between the two groups was 0.00336, of which 39.77% could be explained by observed variables. Emotional and behavioral problems (SDQ) emerged as the largest contributing factor (41.43%, P<0.001), followed by ADHD symptoms (contribution rate: 17.93%, P<0.001) and gender (contribution rate: 3.42%, P<0.001).

It is noteworthy that maternal education level (contribution rate: -19.67%, P = 0.004) and household income (contribution rate: -5.91%, P = 0.019) exhibited significant negative contributions. This indicates that if the only-child group had similar maternal education backgrounds and income distributions to the non-only child group, the anxiety symptom gap between the two groups would be even larger, suggesting that higher socioeconomic status may serve as a protective buffer against anxiety risk for only children.

Taken together, emotional and behavioral problems constitute the core explainable factor underlying the difference in anxiety symptoms between only children and non-only children. However, the variables included in this study explained 39.77% of the difference in anxiety symptoms between only-children and non-only-children, leaving 60.23% of the disparity unexplained by the current model. This may be attributable to unmeasured family process variables, such as parenting styles, parental psychological status, quality of parent–child interactions, marital relationship, and sibling interaction patterns. Future research could employ longitudinal data to further explore the mechanistic pathways through which family structure influences child mental health (**Supplementary Table 5**).

## 4 Discussion

This study, based on large-scale cross-sectional survey data from 67,779 preschool children in Longhua District, Shenzhen, aims to reveal the differences in anxiety symptoms between only children and non-only children in the context of Chinese family structure, as well as the underlying mechanisms. Through multivariate logistic regression and the Fairlie decomposition model, this study not only identified group differences in anxiety symptoms between the two types of children, but also further quantified the relative contributions of various factors to these differences. The results indicated that there was a statistically significant difference in the manifestation of anxiety symptoms between only-child and non-only-child preschool children, although the magnitude of the difference was modest. The Fairlie decomposition revealed that this disparity may be associated with multidimensional factors, including family socioeconomic background and children’s neurobehavioral characteristics. Meanwhile, a portion of the difference remained unexplained by the observed variables, suggesting that the pathways through which family structure affects child mental health are complex and multidimensional in nature.

This study found that the detection rate of anxiety symptoms among only-child preschool children (3.6%) was significantly higher than that among non-only child preschool children (3.3%). Although the magnitude of the difference was small, it reached statistical significance in the context of a large sample, suggesting that children from different family structures may exhibit distinct mental health characteristics, and the only-child group warrants further attention. This finding differs somewhat from the view in some earlier studies that “only children possess psychological advantages due to concentrated resources.” For example, Yang et al., based on evidence from behavioral and brain structure studies, found significant differences between only children and non-only children in creativity and agreeableness, but no consistent disadvantage in mental health-related traits such as emotional stability (23). Falbo and Poston’s study of school-aged children across four provinces in China also confirmed that only children scored higher only on verbal ability tests, with virtually no systematic differences in personality trait assessments compared to those with siblings (24). However, studies conducted in China in recent years have presented a more complex picture. A study of 8,131 Chinese university students showed that the detection rate of psychological symptoms among only children was significantly higher than that among non-only children (25). This evolution in research findings may reflect profound changes in the family environment in Chinese society. In the “post-only-child era,” family investment in children’s education has become more concentrated, and parental expectations and anxiety may also be more focused on the only child, thus creating a family environment characterized by high investment and high expectations (9). Our findings echo recent research on “intensive parenting” and “educational anxiety” among parents. Niu et al.’s survey of 2,932 Chinese parents found that educational anxiety is prevalent among parents, and that family socioeconomic status is significantly negatively correlated with educational anxiety (26). Parenteau et al. further pointed out that parenting styles can amplify or buffer children’s anxiety responses (27). It can be seen that this parenting model of high investment and high expectations may be a double-edged sword, sowing the seeds of anxiety while also promoting development.

In terms of the structure of anxiety symptoms, this study revealed distinct developmental stage characteristics among preschool children. The total mean score on the Spence Preschool Anxiety Scale (SPAS) was 23.110 ± 12.318, indicating that a certain level of anxiety is common among children in this age group. This finding aligns with previous research on preschool children’s emotional development, suggesting that anxiety exhibits certain universal features during the early developmental stage. Regarding symptom structure, separation anxiety (“Not willing to leave you to sleep or leave home for the night,” 1.530 ± 1.310) and physical injury fears (“fear of the dark,” 1.340 ± 1.099) received the highest scores, consistent with the psychological developmental patterns of preschool children, whose attachment relationships are not yet fully differentiated and whose cognitive development remains at the stage of concrete experience. Existing research has indicated that early childhood sensitivity to separation and potential danger is closely related to the establishment of security and the development of imagination, lending strong developmental stage interpretability to these findings (28, 29). A more nuanced finding emerged in the social anxiety dimension, where non-only-children scored slightly higher on some items. This result is not entirely consistent with findings from some previous studies suggesting that sibling relationships play a social support role. (30). One possible explanation is that, within the Chinese family context, sibling relationships not only provide opportunities for social interaction but may also be accompanied by differences in family resource allocation, behavioral comparisons, and differential family roles, all of which may be associated with children’s sensitivity to others’ evaluation. However, as this study did not collect further information on sibling number, birth order, or age spacing, this explanation awaits confirmation in future research (31).

This study further revealed significant differences between the two groups of children in terms of family socioeconomic background and neurobehavioral characteristics. The non-only child group had significantly higher parental education levels (bachelor’s degree or above: mothers 73.5%; fathers 73.5%) and monthly household income (>20,000 RMB: 54.6%) compared to the only-child group (mothers 60.5%; fathers 62.7%; monthly household income 48.3%). This indicates that non-only child families constitute a group with higher socioeconomic status (SES). Meanwhile, non-only children also showed significantly higher rates of ADHD symptom positivity (11.7%) and abnormal SDQ total difficulties scores (20.2%). This pattern is consistent with a large German cohort study, which also found that family SES is closely associated with child mental health, with children from single-parent families exhibiting poorer mental health outcomes across all SDQ dimensions (32). These baseline characteristic differences suggest that any subsequent associations observed should be interpreted with caution within the framework of group heterogeneity.

This study further revealed the heterogeneity of factors influencing anxiety symptoms through logistic regression analysis. In the only-child group, maternal education level was significantly negatively associated with children’s anxiety risk (high school education: OR = 0.751, P<0.01; college education: OR = 0.710, P<0.001). This finding is consistent with previous studies indicating that higher parental education is generally associated with better child mental health outcomes (33). This result is further corroborated by the negative contribution of maternal education level in the Fairlie decomposition (-19.67%), suggesting that, in only-child families, the cognitive resources, health literacy, and parenting capabilities represented by higher maternal education constitute an important protective barrier against anxiety risk. Highly educated mothers may be more adept at recognizing children’s emotional cues, accessing quality parenting resources, and adopting more rational parenting strategies (27).

In the non-only-child group, the association between household income and reduced anxiety risk was more prominent. Compared to the lowest income group, all higher-income subgroups showed significantly lower anxiety risk (OR range:0.426–0.623, all P<0.05), with the strongest association observed in the 30,000–40,000 RMB group (OR = 0.426, P<0.001). This pattern is consistent with a 2025 study based on 69,571 preschool children, which found that the detection rate of anxiety symptoms was significantly lower among children from higher-income families than those from lower-income families (34). This suggests that, in families with multiple children, abundant economic resources may effectively alleviate the resource allocation pressures associated with an increased number of children. However, even when economic conditions improve, the detection rate of anxiety symptoms in non-only-children remains higher than that in only-children (25), indicating that beyond economic resources, non-material factors such as parental time allocation, dispersion of emotional attention, and sibling competition play an irreplaceable role in the mental health of multi-child families.

Within the non-only child group, the influence of household income on children’s anxiety symptoms emerged more prominently. Compared to the lowest income group (<10,000 RMB), all subgroups with a monthly per capita household income exceeding 10,000 RMB exhibited significantly elevated anxiety risk, with the risk peaking in the 30,000–40,000 RMB group (OR = 1.940, *P* < 0.001). This suggests that for families with multiple children, greater economic resources do not necessarily translate into an optimized psychological environment; rather, they may be accompanied by heightened family expectations, accelerated pace of life, and potential competition among siblings for parental attention and resources (35, 36). Despite the group differences, this study also identified common factors associated with anxiety symptoms across both groups. Female gender consistently served as a protective factor in both only children (OR = 0.785) and non-only children (OR = 0.801), a finding consistent with some preschool studies. This may be attributed to the tendency for boys to exhibit greater impulsivity, hyperactivity, or behavioral regulation difficulties in early childhood, characteristics that may expose them to more negative feedback within family or group settings, thereby increasing anxiety risk (37). In contrast, girls generally demonstrate greater stability in emotional expression and behavioral adaptation, resulting in relatively lower anxiety risk (38, 39). Meanwhile, positive ADHD symptoms (OR≈2.53) and abnormal SDQ emotional and behavioral problem scores (OR≈18.28) emerged as the two most prominent risk factors for anxiety symptoms in preschool children. This finding aligns with existing research. For instance, Jensen et al., in a large-sample study using data from the MTA project, found a significant comorbidity between ADHD and anxiety disorders, with ADHD comorbid with anxiety constituting a distinct clinical subtype (40). Similarly, Wakschlag and colleagues’ series of studies demonstrated that early emotional and behavioral problems are important predictors of later internalizing symptoms, such as anxiety and depression, in children (41).

To further explore the underlying sources of group differences, this study employed the Fairlie decomposition model to quantitatively analyze the disparity in anxiety symptoms. The results revealed that among the 0.00336 probability difference in anxiety symptoms between only children and non-only children, 39.77% could be explained by differences in the distribution of observed variables. Emotional and behavioral problems (SDQ) emerged as the largest contributing factor, accounting for 41.43% of the explained difference (*P* < 0.001). This finding quantitatively demonstrates that emotional and behavioral problems are closely associated with childhood anxiety and may be an important factor influencing the level of anxiety in children. It supports the developmental psychopathology perspective, which posits that anxiety often represents a central manifestation of broader difficulties in emotional regulation and behavioral problems (6). ADHD symptoms also contributed substantially to the difference, accounting for 17.93% (*P* < 0.001), underscoring the profound impact of early executive function, attention, and impulse control on emotional health. Inattention and hyperactivity-impulsivity may themselves serve as sources of frustration and negative evaluation, while also impairing children’s capacity to employ cognitive strategies for effectively managing anxiety (42–44). The most intriguing finding lies in the negative contributions of maternal education level (contribution rate: -19.67%, *P* = 0.004) and household income (contribution rate: -5.91%, *P* = 0.019). This result reveals a critical insight: the generally higher socioeconomic status of only-child families actually functions as a “protective buffer.” If the only-child group possessed socioeconomic characteristics similar to those of the non-only child group, the anxiety disparity between the two groups would likely be even larger (45). This finding highlights the necessity of fully considering the moderating role of socioeconomic factors when assessing the impact of family structure on mental health (46).

Based on the findings of this study, to effectively alleviate anxiety symptoms in preschool children and narrow the differences between only children and non-only children, multidimensional and targeted intervention strategies should be developed that take into account the risk characteristics of different family structures. First, a mental health monitoring system centered on SDQ screening should be established and inclusive educational practices promoted. Routine SDQ screening and early intervention mechanisms should be implemented in kindergartens and community healthcare systems (47, 48). Meanwhile, given the significant contribution of ADHD symptoms and the higher detection rate among non-only children, preschool teachers should receive training in special educational needs to provide a supportive learning environment for children with neurodevelopmental diversity (12, 49, 50). Second, differentiated family education guidance should be provided. For only-child families, interventions should focus on alleviating the pressure associated with high maternal education levels, guiding highly educated mothers to develop appropriate educational expectations (51). For non-only-child families, efforts should address sibling rivalry and the dispersion of parental attention to create a harmonious family environment (52). Finally, a supportive environment linking communities, kindergartens, and families should be built. Online mental health platforms can be developed to enhance parents’ eHealth literacy (53), and regular parent-child physical activities should be organized to leverage the protective effect of gender observed in this study (54).

## 5 Limitations and future directions

The present study has the following limitations. First, the cross-sectional design precludes causal inferences regarding the differences in anxiety symptoms between only-children and non-only children; the results of the Fairlie decomposition reflect statistical contributions rather than causal directions. Second, the data were obtained from parental self-report questionnaires, which may be subject to recall bias or social desirability effects, thereby affecting the objectivity of the measurements. Third, the sample was drawn exclusively from a single administrative district in Shenzhen, and the generalizability of the findings to areas with different levels of urbanization or to rural regions requires further validation. Fourth, this study only grouped children according to whether they were only-children or not, without further distinguishing the structural characteristics within non-only-child families, such as sibling number, birth order, or age spacing; therefore, the influence of different sibling relationship patterns on children’s anxiety symptoms could not be evaluated. In addition, a portion of the between-group differences remains unexplained by the included variables. Future research should incorporate micro-environmental variables such as parenting styles and parent-child interaction quality.

## 6 Conclusion

Based on large-scale cross-sectional data from preschool children in Longhua District, Shenzhen, this study employed the Fairlie decomposition model and multivariate logistic regression to reveal significant differences in anxiety symptoms between only-children and non-only children, and quantified the contributions of various factors to these differences. The findings provide important insights for the promotion of child mental health, indicating that to narrow the mental health gap among children from different family structures, attention should not only be paid to early screening and intervention for emotional and behavioral problems, but also to the integration of family socioeconomic factors and children’s neurodevelopmental characteristics. This calls for differentiated family education guidance and multi-sectoral collaborative mental health services tailored to children from different family structures. By systematically analyzing the sources of anxiety symptom differences, this study not only deepens our understanding of the mechanisms through which family structure affects child mental health, but also lays a solid empirical foundation for the development of precise intervention strategies.

## Data Availability

The raw data supporting the conclusions of this article will be made available by the authors, without undue reservation.
