# Supplementary material for: Decomposition and comparative analysis of the differences in anxiety symptoms in preschool children with only and non-only children: a cross-sectional study
Source: Front Psychiatry. 2026 Oct 14;17:1877032. doi: 10.3389/fpsyt.2026.1877032 (PMC13713645; doi:10.3389/fpsyt.2026.1877032)
Supplement: Supplementary file 1 [file Supplementaryfile1.docx]

Supplementary Material

# 1 Supplementary Figures and Tables

## 1.1 Supplementary Figures

**Supplementary Figure 1.** Participant screening flowchart.

**Supplementary Table 1.** SPAS score for preschool children.

| Item | OVERALL  (x±s) | ONLY CHILD(x±s) | NON- ONLY CHILD(x±s) |
| --- | --- | --- | --- |
| Generalized anxiety |  |  |  |
| Q1:It's hard not to worry him/her | 0.994±0.794 | 0.958±0.792 | 1.075±0.792 |
| Q4:Tense, uneasy, irritable due to worry | 0.670±0.752 | 0.640±0.745 | 0.710±0.766 |
| Q8:I can't sleep well because of my fear | 0.508±0.683 | 0.511±0.686 | 0.501±0.675 |
| Q14:I spend most of the day worrying about all kinds of things | 0.318±0.567 | 0.321±0.570 | 0.311±0.561 |
| Q28:Comfort is also sought when necessarily | 0.874±0.864 | 0.857±0.860 | 0.910±0.872 |
| Social anxiety |  |  |  |
| Q2:Worried that you would do stupid things in front of others | 0.781±0.786 | 0.746±0.772 | 0.858±0.812 |
| Q5:Afraid of asking for adult help (such as a kindergarten teacher) | 1.011±0.908 | 0.991±0.901 | 1.055±0.921 |
| Q11:Fear of meeting strangers or talking to unfamiliar people | 0.845±0.955 | 0.817±0.937 | 0.905±0.991 |
| Q15:Afraid to speak or speak in front of the whole class (kindergarten group) | 2.48±0.656 | 2.460±0.665 | 2.540±0.631 |
| Q19:Worried that you will do embarrassing things in front of others | 0.661±0.775 | 0.648±0.768 | 0.689±0.791 |
| Q23:Fear of attending activities with the other children | 0.358±0.625 | 0.362±0.627 | 0.350±0.619 |
| Separation anxiety |  |  |  |
| Q6:Not willing to leave you to sleep or leave home for the night | 1.530±1.310 | 1.470±1.287 | 1.650±1.349 |
| Q12:Worried that something bad will happen to the parents | 0.480±0.718 | 0.474±0.713 | 0.493±0.730 |
| Q16:Worried that something bad might happen to you (like getting lost or being abducted) and that you wouldn't see you again | 0.800±0.838 | 0.788±0.835 | 0.826±0.842 |
| Q22:It can be frustrating to put him or her in a nursery or leave them with temporary caregivers | 0.521±0.728 | 0.508±0.715 | 0.551±0.755 |
| Q25:Do the nightmare of separation from you | 0.411±0.649 | 0.409±0.652 | 0.416±0.642 |
| Physical injury fears |  |  |  |
| Q7: fear of height | 0.937±0.990 | 0.909±0.975 | 1.001±1.021 |
| Q10:Fear of staying in crowded or enclosed places | 0.715±0.929 | 0.715±0.926 | 0.714±0.936 |
| Q13:Fear of lightning | 1.110±0.977 | 1.100±0.969 | 1.150±0.992 |
| Q17:I felt very nervous when crossing the road | 0.550±0.732 | 0.560±0.740 | 0.510±0.714 |
| Q20:Fear of bugs and / or spiders | 1.293±1.121 | 1.291±1.112 | 1.296±1.141 |
| Q24:I'm afraid of dogs | 0.993±1.080 | 1.008±1.084 | 0.960±1.070 |
| Q26:fear of the dark | 1.340±1.099 | 1.310±1.088 | 1.420±1.117 |
| Obsessive-compulsive disorder |  |  |  |
| Q3:Keep checking if you have things done (like the door off; the tap off) | 0.660±0.844 | 0.673±0.852 | 0.630±0.825 |
| Q9:Wash your hands over and over again every day | 0.684±0.926 | 0.694±0.929 | 0.662±0.920 |
| Q18:Things must be placed in a fixed order or position to prevent bad things from happening | 0.817±0.928 | 0.820±0.925 | 0.811±0.936 |
| Q21:Some bad or stupid thoughts or images come back over and over again | 0.465±0.658 | 0.464±0.657 | 0.467±0.662 |
| Q27:You have to always think of something special (like a number or word) to prevent bad things from happening | 0.303±0.565 | 0.317±0.576 | 0.272±0.539 |
| Total score | 23.110±12.318 | 22.820±12.536 | 23.740±11.799 |

**Supplementary Table 2.** Distribution of the variables in only and non-only children respondents.

| VARIABLE | ONLY CHILD[n(%)] | NON- ONLY CHILD[n(%)] | *x*^2^ | P |
| --- | --- | --- | --- | --- |
| SPAS |  |  | 4.893 | 0.027 |
| ≥48 | 1671（3.6） | 690（3.3） |  |  |
| ＜48 | 44894（96.4） | 20524（96.7） |  |  |
| Gender |  |  |  |  |
| Male | 24468（52.5） | 11706（55.2） | 40.651 | ＜0.001 |
| Female | 22097（47.5） | 9508（44.8） |  |  |
| Maternal education |  |  | 1303.931 | ＜0.001 |
| Junior high school or lower | 8044（17.3） | 2107（9.9） |  |  |
| High school | 10337（22.2） | 3517（16.6） |  |  |
| College | 27018（58.0） | 14505（68.4） |  |  |
| Master’s degree or above | 1166（2.5） | 1085（5.1） |  |  |
| Paternal education |  |  | 840.846 | ＜0.001 |
| Junior high school or lower | 7086（15.2） | 2004（9.4） |  |  |
| High school | 102744（22.1） | 3633（17.1） |  |  |
| College | 27088（58.2） | 14153（66.8） |  |  |
| Master’s degree or above | 2117（4.5） | 1424（6.7） |  |  |
| Monthly per capita household income |  |  | 305.713 | ＜0.001 |
| <10,000RMB | 7691（16.5） | 2633（12.4） |  |  |
| 10,000–20,000RMB | 16399（35.2） | 7003（33.0） |  |  |
| 20,000–30,000RMB | 9675（20.8） | 4981（23.5） |  |  |
| 30,000–40,000RMB | 5226（11.2） | 2833（13.4） |  |  |
| >40,000RMB | 7574（16.3） | 3764（17.7） |  |  |
| ADHD symptoms |  |  | 154.332 | ＜0.001 |
| Yes | 4022（8.6） | 2475（11.7） |  |  |
| No | 42543（91.4） | 18739（88.3） |  |  |
| SDQ |  |  | 133.400 | ＜0.001 |
| 0-13 | 38645（83.0） | 16934（79.8） |  |  |
| 14-16 | 4726（10.1） | 2779（13.1） |  |  |
| 17-40 | 3194（6.9） | 1501（7.1） |  |  |

**Supplementary Table 3.** Distribution of variables among survey respondents the presence or absence of anxiety symptoms.

| VARIABLE | The presence of anxiety symptoms | | | | The absence of anxiety symptoms | | | |
| --- | --- | --- | --- | --- | --- | --- | --- | --- |
|  | ONLY CHILD | NON- ONLY CHILD | *x^2^* | P | ONLY CHILD | NON- ONLY CHILD | *x^2^* | P |
|  | [n(%)] | [n(%)] |  |  | [n(%)] | [n(%)] |  |  |
| Gender |  |  | 2.285 | 0.131 |  |  | 38.055 | ＜0.001 |
| Male | 805（48.2） | 356（51.6） |  |  | 23663（52.7） | 113500（55.3） |  |  |
| Female | 866（51.8） | 334（48.4） |  |  | 21231（47.3） | 9174（44.7） |  |  |
| Maternal education |  |  | 25.358 | ＜0.001 |  |  | 1278.261 | ＜0.001 |
| Junior high school or lower | 434（26.0） | 130（18.8） |  |  | 7610（17.0） | 1977（9.6） |  |  |
| High school | 376（22.5） | 132（19.1） |  |  | 9961（22.2） | 3385（16.5） |  |  |
| College | 827（49.5） | 403（58.4） |  |  | 26191（58.3） | 14102（68.7） |  |  |
| Master’s degree or above | 34（2.0） | 25（3.6） |  |  | 1132（2.5） | 1060（5.2） |  |  |
| Paternal education |  |  | 15.3 | 0.002 |  |  | 825.628 | ＜0.001 |
| Junior high school or lower | 346（20.7） | 105（15.2） |  |  | 6740（15.0） | 1899（9.3） |  |  |
| High school | 415（24.8） | 156（22.6） |  |  | 9859（22.0） | 3477（16.9） |  |  |
| College | 848（50.7） | 392（56.8） |  |  | 26240（58.4） | 13761（67.0） |  |  |
| Master’s degree or above | 62（3.7） | 37（5.4） |  |  | 2055（4.6） | 1387（6.8） |  |  |
| Monthly per capita household income |  |  | 8.393 | 0.078 |  |  | 302.242 | ＜0.001 |
| <10,000RMB | 442（26.5） | 160（23.2） |  |  | 7249（16.1） | 2473（12.0） |  |  |
| 10,000–20,000RMB | 573（34.3） | 230（33.3） |  |  | 15826（35.3） | 6773（33.0） |  |  |
| 20,000–30,000RMB | 292（17.5） | 147（21.3） |  |  | 9383（20.9） | 4834（23.6） |  |  |
| 30,000–40,000RMB | 162（9.7） | 57（8.3） |  |  | 5064（11.3） | 2776（13.5） |  |  |
| >40,000RMB | 202（12.1） | 96（13.9） |  |  | 7372（16.4） | 3668（17.9） |  |  |
| ADHD symptoms |  |  | 2.293 | 0.130 |  |  | 162.639 | ＜0.001 |
| Yes | 387（23.2） | 180（26.1） |  |  | 3635（8.1） | 2295（11.2） |  |  |
| No | 1284（76.8） | 510（73.9） |  |  | 41259（91.9） | 18229（88.8） |  |  |
| SDQ |  |  | 2.916 | 0.233 |  |  | 278.416 | ＜0.001 |
| 0-13 | 556（33.3） | 220（31.9） |  |  | 38089（84.8） | 16714（81.4） |  |  |
| 14-16 | 351（21.0） | 167（24.2） |  |  | 4375（9.7） | 2612（12.7） |  |  |
| 17-40 | 764（45.7） | 303（43.9） |  |  | 2430（5.5） | 1198（5.8） |  |  |

**Supplementary Table 4.** Logistic regression results for sociodemographic characteristics associated with anxiety symptoms.

| VARIABLE | OVERALL | | | ONLY CHILD | | NON- ONLY CHILD | |
| --- | --- | --- | --- | --- | --- | --- | --- |
|  | OR | | 95%CI | OR | 95%CI | OR | 95%CI |
| Gender |  | |  |  |  |  |  |
| Male | 1 | |  | 1 |  | 1 |  |
| Female | 1.240*** | （1.139,1.350） | | 1.224*** | （1.106,1.354） | 1.276** | （1.090,1.493） |
| Maternal education |  | |  |  |  |  |  |
| Junior high school or lower | 1 | |  | 1 |  | 1 |  |
| High school | 0.761*** | | （0.663,0.873） | 0.751** | （0.641,0.881） | 0.771 | （0.584,1.018） |
| College | 0.702*** | | （0.611,0.805） | 0.710** | （0.605,0.833） | 0.690** | （0.526,00905） |
| Master’s degree or above | 0.658** | | （0.478,0.906） | 0.681 | （0.452,1.028） | 0.653 | （0.386,1.103） |
| Paternal education |  | |  |  |  |  |  |
| Junior high school or lower | 1 | |  | 1 |  | 1 |  |
| High school | 1.058 | | （0.919,1.218） | 1.052 | （0.894,1.238） | 1.074 | （0.0.810,1.423） |
| College | 0.963 | | （0.834,1.112） | 0.990 | （0.838,1.170） | 0.906 | （0.682,1.205） |
| Master’s degree or above | 1.024 | （0.785,1.335） | | 1.056 | （0.762,1.461） | 0.970 | （0.608,1.548） |
| Monthly per capita household income |  |  | |  |  |  |  |
| <10,000RMB | 1 |  | | 1 |  | 1 |  |
| 10,000–20,000RMB | 0.684*** | （0.609,0.768） | | 0.707*** | （0.617,0.810） | 0.623*** | （0.499,0.779） |
| 20,000–30,000RMB | 0.646*** | （0.561,0.743） | | 0.651*** | （0.550,0.771） | 0.622*** | （0.480,0.806） |
| 30,000–40,000RMB | 0.611*** | （0.513,0.727） | | 0.713** | （0.582,0.873） | 0.426*** | （0.409,0.741） |
| >40,000RMB | 0.585*** | （0.498,0.687） | | 0.595*** | （0.491,0.721） | 0.550*** | （0.409,0.741） |
| ADHD symptoms |  |  | |  |  |  |  |
| Yes | 1 |  | | 1 |  | 1 |  |
| No | 0.395*** | （0.355,0.439） | | 0.378*** | （0.332,0.430） | 0.423*** | （0.351,0.510） |
| SDQ |  |  | |  |  |  |  |
| 0-13 | 1 |  | | 1 |  | 1 |  |
| 14-16 | 9.573*** | （7.956,11.519） | | 9.466*** | （7.558,11.854） | 9.902*** | （7.139,13.736） |
| 17-40 | 18.278*** | （14.539,22.979） | | 19.586*** | （14.897,25.751） | 15.314*** | （10.019,23.409） |

**Supplementary Table 5.** The Fairlie decomposition Model of Anxiety Symptom Status in Preschool Children with Only and Non-only Children.

| Terms of decomposition | | | Anxiety symptoms | |
| --- | --- | --- | --- | --- |
| Difference | | | 0.00335963 | |
| Explained (%) | | | 0.0013362（39.77） | |
| Non-explained (%) | | | 0.00202343（60.23） | |
| Contribution to differences | B | P | Contribution (%) | [95%CI] |
| Explained |  |  |  |  |
| Gender | 0.0001148 | ＜0.001 | 3.417042948 | (0.0000512 ,0.0001783) |
| Maternal education | -0.0006609 | 0.004 | -19.6718091 | (-0.0011055,-0.0002163) |
| Paternal education | 0.0000599 | 0.738 | 1.78293443 | (-0.000291,0.0004109) |
| Monthly per capita household income | -0.0001987 | 0.019 | -5.914341758 | (-0.0003642,-0.0000333) |
| ADHD symptoms | 0.0006023 | ＜0.001 | 17.9275694 | (0.0004068,0.0007978) |
| SDQ | 0.001392 | ＜0.001 | 41.43313401 | (0.0012561,0.0009303) |
